# Photon Counting Imaging with an Electron-Bombarded Pixel Image Sensor

**DOI:** 10.3390/s16050617

**Published:** 2016-04-28

**Authors:** Liisa M. Hirvonen, Klaus Suhling

**Affiliations:** Department of Physics, King’s College London, Strand, London WC2R 2LS, UK; liisa.2.hirvonen@kcl.ac.uk

**Keywords:** photon counting, electron-bombarded sensor, single photon detection, low light level imaging, EBCCD, EBCMOS

## Abstract

Electron-bombarded pixel image sensors, where a single photoelectron is accelerated directly into a CCD or CMOS sensor, allow wide-field imaging at extremely low light levels as they are sensitive enough to detect single photons. This technology allows the detection of up to hundreds or thousands of photon events per frame, depending on the sensor size, and photon event centroiding can be employed to recover resolution lost in the detection process. Unlike photon events from electron-multiplying sensors, the photon events from electron-bombarded sensors have a narrow, acceleration-voltage-dependent pulse height distribution. Thus a gain voltage sweep during exposure in an electron-bombarded sensor could allow photon arrival time determination from the pulse height with sub-frame exposure time resolution. We give a brief overview of our work with electron-bombarded pixel image sensor technology and recent developments in this field for single photon counting imaging, and examples of some applications.

## 1. Introduction

Photon counting imaging is a well-established low light level imaging technique where an image is assembled from individual photons whose position is recorded during the detection process, usually with a position-sensitive sensor (*i.e.*, a camera). In astronomy, photon counting imaging technology was originally introduced due to its sensitivity, and continues to be used on both ground- and space-based observatories, particularly in the UV [1,2,3,4]. Other advantages of photon counting imaging include linearity, high dynamic range, high sensitivity, zero read-out noise and well-defined Poisson statistics, and photon counting imaging is now finding applications in diverse fields of science, such as fluorescence spectroscopy and microscopy, LIDAR, optical tomography and quantum cryptography, as reviewed recently [5,6,7,8].

The light detection capability of solid state sensors is based on their ability to convert photons, via electron-hole pair generation, into an electronic signal that can be read out. Despite recent developments in these sensors, especially in CMOS technology which now allow megapixel resolution and up to MHz frame rates [9], these detectors are still not sensitive enough to detect single photons without amplification [10]. Several methods have been developed to produce a detectable output signal from the incoming single photons. Traditionally, microchannel plate (MCP) image intensifiers have been used to amplify the signal, and intensified CCDs (ICCDs), which combine a photon counting MCP-intensifier with a CCD camera in one package, have been commercialised. In these devices, a single photon creates a photoelectron which creates secondary electrons as it travels through the MCPs, before the electrons are converted back into photons with a phosphor screen, coupled to the CCD with by a fibre optic taper or a lens. Electron-multiplying CCDs (EMCCDs) where the signal is amplified in a gain register placed between the shift register and the output amplifier, are also single photon sensitive and commercially available, and an EMCMOS concept has been demonstrated [11].

Single photon detection is also possible with electron-bombarded (EB) sensors, where a photocathode is placed in front of the sensor, and a single photoelectron is accelerated through a high voltage directly into a solid state sensor. EB sensors are conceptually similar to old silicon intensified target television cameras, and have found applications in microscopy [12,13] and biological low-light imaging [14,15,16], optical spectroscopy [17] and radiography [18,19]. The advantages of EB sensors over intensified camera systems include reduced sensor size and weight, increased sensitivity and dynamic range, faster response time, and better contrast and resolution. They require a high voltage of several kV between the photocathode and the CCD sensor in vacuum, and backscattered photoelectrons can be detected on the low energy side of the pulseheight distribution, making it asymmetric [20]. Note that manufacture of such a device requires skilful incorporation of a wire-bonded silicon chip into a vacuum tube enclosure with cleanliness, low outgassing and vacuum bake requirements. In contrast to intensifiers, it neither requires microchannel plates, nor a phosphor screen.

The EB concept has also been utilised in point detectors. In a hybrid photodetector (HPD), a photocathode is placed in front of an avalanche photodiode (APD) and single photons are accelerated into the APD [21,22]. To provide both spatial and timing resolution, HPD arrays have been built and demonstrated [23] and linear 16 HPDs are now commercially available, e.g., for spectrally-resolved fluorescence lifetime measurements.

We note here that recent developments in single photon avalanche diode (SPAD) detectors, which can be manufactured in large arrays using CMOS technology, show great promise as an alternative to vacuum-based detector technology. They simultaneously deliver single photon sensitivity and picosecond timing resolution in tens of thousands of pixels, and have the potential to significantly advance time-resolved fluorescence microscopy and other fields, see Section 5 for more details.

## 2. EB-Technology

### 2.1. How EB-Sensors Work

**Figure 1:**
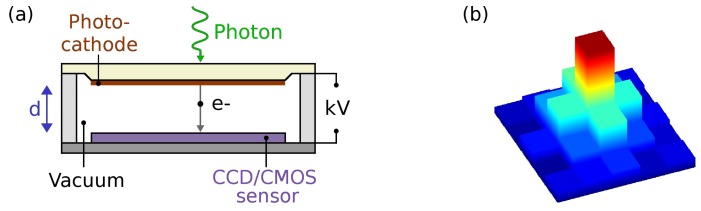
(**a**) Schematic diagram of an electron bombarded sensor. A photon impinging on the photocathode liberates a photoelectron, e^−^. Using a large potential difference of several kV, the photoelectron is accelerated over a distance *d* into the sensor, where it creates electron-hole pairs; in silicon, one electron-hole pair for each 3.7 eV depending on conditions such as temperature and impurities; (**b**) Schematic of typical photon event charge distribution in the sensor. The event covers several pixels, with a high central peak and small wings.

An EB sensor combines a photocathode with a silicon solid state CCD or CMOS sensor under vacuum, as illustrated in Figure 1a, such that a single photoelectron, ejected from the photocathode by a photon, is directly accelerated into the sensor by a high voltage of several kVs [24]. In the sensor, the electron creates a well-defined number of electron-hole pairs (around one electron-hole pair per 3.7 eV in silicon [25]) and consequently the pulse height distribution of these devices is narrow and strongly dependent on the acceleration voltage.

EB-sensors are usually back-thinned (as the front side electronic layers prevent any low energy particle detection), leading to charge sharing between pixels; during the diffusion of the electrons from the back of the sensor to the front the charge spills over into adjacent pixels, and the photon events typically have a sharp central peak and small wings (Figure 1b). Centroiding methods can be employed to find the photon event location with sub-pixel accuracy; in this case the resolution is governed by the proximity focussing principle [26] (*i.e.*, the photoelectron trajectories from the photocathode to the sensor over a small, typically ∼1 mm gap). The backscatter in our EB-sensor is small [20], and, in general, EB-sensors have better contrast and resolution compared to MCP intensified systems, where backscattering of electrons degrades the image quality [27].

Besides photon events, another type of event detected with EB-sensors are ion events, which are caused by an electron hitting a residual gas molecule inside the imperfect vacuum. The gas molecule is ionised and accelerated towards the photocathode, where it causes secondary emission of electrons, and bright, large events when the electrons hit the sensor. In MCP-based intensifiers, the MCPs are placed in a chevron arrangement, *i.e.,* with a small bias angle, to minimise this type of ion feedback, but in EB-sensors, there is no such barrier and a free line of sight between the photocathode and the sensor. These ion events can usually be discarded during data processing, as their large size and brightness allows them to be easily differentiated from the photon events.

Using a simple model, the gain of an EB-sensor is determined by [27,28]

(1) $$EB_{gain}=\frac{V-V_{th}}{W}$$

where *V* is the potential difference between the cathode and the sensor, $V_{th}$ is the threshold voltage, and *W* is the energy needed to create one electron-hole pair in the sensor. More detailed gain models have been devised, taking backscattered photoelectrons into account [27]. For silicon, *W* is ∼3.7 eV, depending on the local conditions [25,29]. The sensor is covered by a layer of aluminium, and thus only electrons with energy above a threshold energy $eV_{th}$ will be detected. The gain is thus strongly dependent on the acceleration voltage, leading to a narrow pulse height distribution. The variance in gain, $\sigma^{2}$, is expressed as

(2) $$\sigma^{2}=F\times EB_{gain}$$

where F is the Fano factor (0.12 for silicon) [30].

The time-of-flight *τ* of a photoelectron with mass *m* and charge *q* from the photocathode to the sensor is given by

(3) $$\tau =\frac{d}{\sqrt{2V}}\sqrt{\frac{m}{q}}$$

where *d* is the distance between the cathode and CCD and *V* is the potential. For a typical potential *V* of a few kV and a typical distance *d* around 1 mm, the time of flight for electrons is few tens of ps.

### 2.2. EBCCD Cameras

The first reports in the literature characterising EBCCD cameras appear in the 1980s [31,32,33,34]. They were originally developed for their high signal-to-noise ratio under low light levels [28] which was deemed a considerable advantage for astronomical applications [35]. EBCCDs were also developed for military night vision applications [27]. The first commercially available EBCCD was made by Hamamatsu in 2000, and offered 1024 × 2014 pixels, operating voltage of 6–8 kV and a 3 Hz maximum frame rate [36]. Other EBCCD developments have reported frame rates up to 200 Hz [37], and maximum acceleration voltages of 14 kV and 15 kV [18,38,39]. Intensified EBCCDs, comprising a MCP between the photocathode and the CCD, have also been developed [40].

### 2.3. EBCMOS Cameras

With CMOS cameras each pixel, or row of pixels, has its own amplification and read-out electronics, and can thus achieve faster frame rates than CCD cameras [10]. CMOS sensor technology has developed at a rapid pace over the past two decades, now replacing CCD cameras in consumer electronics and also in scientific research. EBCMOS sensors were first developed for night vision devices in the military [41,42]. For scientific research, the development of EBCMOS cameras originated from applications in particle physics, and Mimosa 5 was demonstrated in 2007, with 1024 × 1024 pixels, 40 Hz frame rate and operating voltage of 6–10 kV [43]. A number of other sensors have been developed [44,45], with the latest development offering a 500 Hz frame rate [46].

A CMOS pixel read-out chip, developed in CERN for particle physics applications, the MediPix2/TimePix ASIC with 256 × 256 pixels and 55 μm pixel size, has been combined with EB concept for single photon detection [47]. Using a clock of 100 MHz and a parallel readout the entire chip can be read out in 266 μs, which makes frame rates of over 3000 fps possible [48].

### 2.4. Photon Arrival Timing

Unlike MCP-intensifiers, EB sensors cannot directly provide photon arrival timing. Point detectors (HPDs) are often combined with TCSPC timing electronics based on a time-to-amplitude converter (TAC) and used for photon arrival timing, for example, in fluorescence lifetime imaging scanning fluorescence microscopy [21,22], but with pixel image sensors photon arrival timing is less straightforward. Although the Medipix/Timepix chips are in principle capable of high timing resolution, their main drawback is 266 μs frame read-out time which limits the global count rate, and they find more applications in photon counting imaging where the arrival timing of the photons is not required and the photons can be accumulated in each pixel before the frame readout.

With EB-sensors, it could be possible to exploit the dependency between the photon event brightness and the acceleration voltage for photon arrival timing [20,49,50]. By varying the voltage in time, in a similar fashion to varying the voltage on the deflector plates in a cathode ray oscilloscope or a streak camera, the photon event height in the sensor corresponds to the photon arrival time at the photocathode. Thus, by converting the arrival time into an amplitude, each pixel is used as a photoelectronic TAC, see Section 3.3. This approach could parallel-process the arrival time of photons in each pixel of the image simultaneously. This kind of time-tagging is not possible with MCP-based intensified CCD cameras due to the broad pulse height distribution of MCPs [24].

## 3. Experimental Characterisation

### 3.1. Single Photon Events & Centroiding

**Figure 2:**
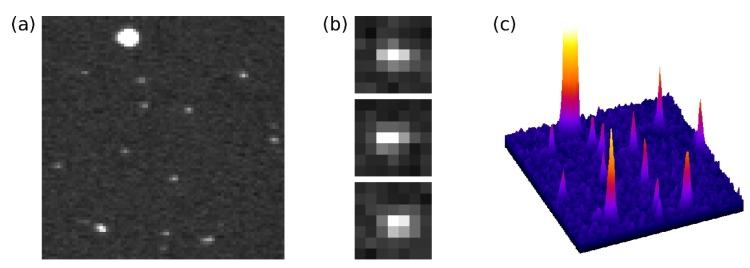
(**a**) 80 × 80 pixel area of a frame with single photon events as detected with a Hamamatsu electron-bombarded CCD at 8 kV acceleration voltage and maximum read-out gain. A bright, large ion event can be seen near the top edge; (**b**) Enlarged areas of three photon events; (**c**) 3D representation of the area in (**a**).

Typical single photon events detected with a Hamamatsu C7190-13 EBCCD at the maximum 8 kV acceleration voltage and maximum read-out gain are shown in Figure 2. The central peak is high with small wings; during the diffusion of the electrons from the back of the sensor to the front, the charge spills over into adjacent pixels, although the pixel’s full well capacity is not reached [20]. Brighter, larger ion events are also detected. Single photon events detected with EBCMOS cameras are reported to be very similar to EBCCD photon events (see, for example, Figure 3 in [46]).

In EB pixel image sensors, the pixel’s potential wells can be filled by the electrons created by only a few photons. For this reason, EB sensors usually have large pixels [51], to facilitate collection of many photons per pixel in analogue fashion. However, for single photon counting applications, a maximum of one photon is collected per pixel per frame, and with photon counting approaches where the photon events cover an area bigger than one pixel, the resolution of the image does not need to be limited by the sensor pixel size. A characteristic feature of this method is the possibility of employing a centroiding technique, where the position of a photon event can be determined with sub-pixel accuracy [52,53,54,55,56]. With EB-sensors, centroiding can be used to recover the resolution lost in the electron diffusion process, and the resolution of the image is then limited by the photoelectron trajectories from the photocathode to the sensor, governed by the proximity focussing principle [26].

**Figure 3:**
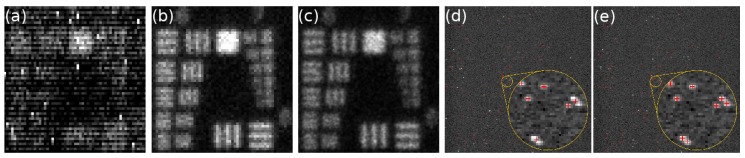
(**a**–**c**) Images of a USAF test pattern obtained by photon counting imaging with a Hamamatsu EBCCD: (**a**) sum of 30,000 frames; (**b**) 1-pixel centroiding; (**c**) 1/5-pixel centroiding. With 1 pixel centroiding, the photon event is assigned to the to the center pixel of the event and the edges are ignored. With 1/5 pixel centroiding, each pixel is divided into 5 × 5 subpixels, and each photon event is assigned a sub-pixel according to the centroid position calculated by the sub-pixel localisation algorithm; (**d**–**e**) A raw frame of USAF data with photon positions localised with super-resolution software marked with red crosses. Overlapping events that are normally counted as one event (**d**) are resolved with multi-emitter fitting analysis (**e**) [58].

The algorithms employed for event centroiding in photon counting imaging were originally developed for implementation in hardware and are based on a simple center-of-mass calculation [52]. The centroiding is nowadays done in software but the algorithms employed in photon counting imaging are still usually simple, one-iteration algorithms [3]. Recently, we have applied iterative algorithms developed for super-resolution fluorescence microscopy for centroiding of photon events, and found that these algorithms yield excellent results for both MCP-intensified camera systems [57] and EBCCDs (Figure 3a–c) [58], providing efficient photon event recognition, low fixed pattern noise and excellent localisation results. Moreover, multi-emitter fitting algorithms–developed for super-resolution microscopy to separate fluorescent emitters whose point-spread functions overlap partially–allow separation of overlapping photon events with EBCCDs, see Figure 3d,e, an important aspect to facilitate an increased count rate and shorter acquisition times.

### 3.2. Pulse Height Distribution

**Figure 4:**
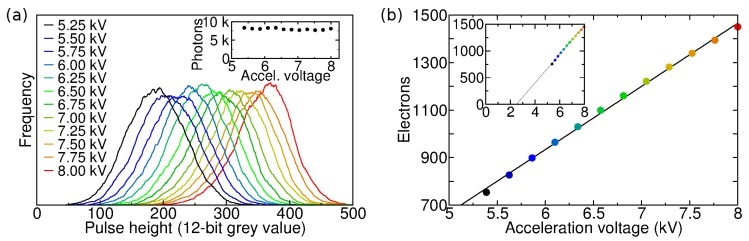
(**a**) Photon event pulse height distributions for different acceleration voltage settings, measured with a Hamamatsu EBCCD. The count rate and number of acquired frames was the same for each setting, resulting in the integral of the distributions being similar (inset); (**b**) Mean pulse height in electrons versus acceleration voltage. A straight line fit yields a gradient of 266 e/kV, from which an electron-hole pair generation energy in silicon, 3.76 eV, can be obtained. The inset shows the same data with extended data range; the threshold voltage 2.5 kV is at *y* = 0.

The pulse height distribution of an EB sensor can provide information about the electron-hole generation process in the sensor. The pulse height distributions of a Hamamatsu C7190-13 EBCCD were measured for different acceleration voltages (Figure 4a). The slight asymmetry is probably due to backscattered photoelectrons [20]. The mean pulse height was plotted against the acceleration voltage (Figure 4b). A straight line fit according to Equation (1) yields a gradient of 266 e/kV, and the energy needed for the creation of one electron-hole pair in silicon can be determined from the inverse gradient: 3.76±0.05 eV. A mean threshold voltage of 2.5±0.1 kV for this device can be found from the x-intercept (Figure 4b); only electrons with an energy above this threshold are detected due to the aluminium layer protecting the CCD. According to Equation (1), this yields a maximum gain of ∼1500 at 8 kV for this step [20,50].

### 3.3. Photon Arrival Timing

**Figure 5:**
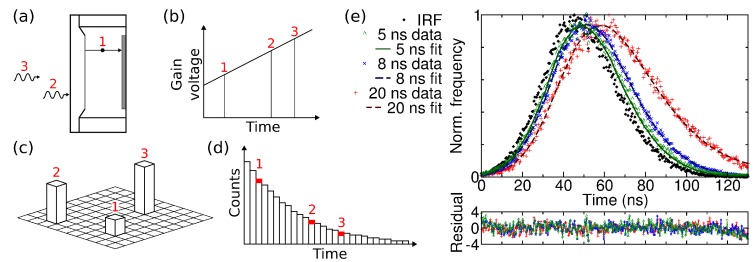
(**a**–**d**) Schematic of proposed gain sweep scheme. Photons arriving at different times during the CCD exposure (**a**) experience different gain voltage which is linearly swept in time (**b**); The pulse heights (**c**) can be converted to arrival times and added to the arrival time histogram (**d**); The gain sweep is repeated many times to build up a histogram for each pixel of the image; (**e**) Simulated fluorescence decays obtained from frames acquired at different acceleration voltages, and the instrument response function (IRF) obtained from measurement with the highest voltage (simulated time 0). The arrival time of each photon was found from its pulse height. Single-exponential fits to the decays yield decay times of 4.72, 8.78 and 20.27 ns for the 5, 8 and 20 simulated decays, respectively [20].

Since the photon event brightness is strongly dependent on the acceleration voltage, it could be possible to use a gain sweep during exposure for photon arrival timing. By sweeping the voltage, each EBCCD frame would consist of photon events of different heights, which represent the arrival time after an excitation pulse, see Figure 5a–d. The frame is read into a computer, where it is analysed and the pulse height and pixel coordinates are stored. By repeating this process many times, *i.e.*, acquiring and analysing many frames, a histogram of photon arrival times is built up in each pixel of the image. This method could be used to measure fluorescence decays. The fluorescence decay can be a function of viscosity, temperature, pH, ion or oxygen concentrations, glucose, refractive index or polarity, and of interaction with other molecules, e.g., via Förster resonance energy transfer [59]. The fluorescence decay is characterised by the fluorescence lifetime, which is the average time a fluorophore remains in the excited state after excitation. By determining the fluorescence lifetime in each pixel of an image, via fluorescence lifetime imaging (FLIM), image contrast according to the fluorescence lifetime is obtained.

As there are currently no devices that allow the acceleration voltage to be changed during exposure, linear sweeps from high to low voltage with 50 ns sweep time and 5, 8 and 20 ns decay times were simulated to test the determination of photon arrival time from the pulse height [20]. The decays were simulated by acquiring sets of frames with Hamamatsu C7190-13 EBCCD at different acceleration voltages. A number of frames from each data set were combined in such a proportion as to yield exponential decays. The photons were thus distributed as if they had arrived at different times during a gain voltage sweep. The frames were processed as a single data set, where the pulse height of each photon was converted to arrival time and added to an arrival time histogram. This was done with the aid of Figure 4b which is effectively a calibration curve to convert photon event brightness into an acceleration voltage (which varies linearly in time). The key point here is not the linearity of the sweep, but the stability and reproducibility of the calibration of brightness versus time. The arrival time histograms, shown in Figure 5e, are in fact the fluorescence decay curves. The histograms were fitted with single-exponential decay law (Figure 5e, lines) using iterative reconvolution and the 8 kV pulse height distribution as an instrumental response function (Figure 5e, black diamonds). This yields decay times of 20.27, 8.78 and 4.72 ns for the 20, 8 and 5 ns simulated decays with chi-squared values of 1.03, 1.08 and 1.31. The residuals are flat, without any systematic deviations. The simulation shows that photon arrival times can be obtained from the photon event pulse heights.

## 4. Some Applications of EB-Sensors

EB pixel image sensors were developed for high-resolution imaging with high signal-to-noise ratio at extremely low light level, and the low light level imaging capability has been utilised in night vision applications [27,41,42]. The single photon detection capabilities were first used in particle physics applications, for example, in observing neutrino interactions at CERN [39], and in astronomical applications [35]. Although recent developments in EMCCDs and sCMOS sensors have meant that the sensitivity advantage has disappeared [10], EB pixel image sensors continue to find applications in particle physics and in life science imaging.

In life sciences, the sensitivity and low noise of EB-sensors allows imaging of weak luminescence signals that are difficult to detect with other sensors. For microscopy applications, EB-sensors have been demonstrated to be suitable for imaging cells at low light level [20,43,45,50], and EBCMOS cameras have been used for tracking of multiple single-emitters [60]. EBCCDs have been used to visualise protein interactions in plant and animal cells and in tissues with subcellular resolution using bioluminescence resonance energy transfer (BRET) imaging [14], and EBCMOS cameras have been applied to marine bioluminescence imaging [61]. EBCCDs have also been evaluated for use in combination with a spectrometer, where high sensitivity in combination with high spatial resolution is required [17].

EB-sensors could also find applications in clinical use. In X-ray digital radiography and computed tomography, the low light level imaging capability of an EBCCD allows the reduction of the irradiation dose to the patient [18,19]. EBCCDs have also been evaluated for visualising stimulated functional brain areas during surgery [16].

## 5. Discussion

EB-sensors are more compact, smaller and lighter than intensified camera systems. The single photoelectrons are accelerated directly into the solid state sensor without MCP intensification and without being converted back into light on a phosphor screen, which is then imaged. With GaAsP photocathodes, the quantum efficiency can reach ∼50%, and unlike MCP detectors, EB sensors have a fill factor or open area ratio of ∼100%. The device lifetime is limited by the damage done to the chip by the high energy electrons striking it and producing x-rays, and ion events reduce the photocathode lifetime. However the relatively small volume and surface area compared to traditional image intensified tubes increase the photocathode lifetime. A lifetime of 10^12^ cnts/mm^2^ has been quoted for EBCCDs, an order of magnitude longer than MCP devices [62].

The photon event brightness in EB pixel image sensors is strongly dependent on the acceleration voltage, and as the photon events typically cover an area of a few pixels, resolution lost in the detection process can be recovered by photon event centroiding—both one-iteration centre-of-mass [20] and iterative fitting [58] algorithms have been shown to produce excellent results. The local count rate is given by the frame rate of the camera; EBCMOS cameras with 500 frames per second have been described [46], and 1000 Hz planned. The global count rate depends on the number of pixels in the sensor, and as both CMOS and CCD sensors can be manufactured in large, megapixel arrays, the detection of hundreds or thousands of photons per frame is possible. The imaging speed is usually limited by the CCD or CMOS read-out time, and with photon event centroiding, the localisation time depends on the complexity of the algorithm [57].

However, despite the many applications and advantages of the ideal EBCCD, and being commercially available for over 15 years, there have been drawbacks, such as low frame rates of a few Hz and artefacts in the images, and it seems the development of these sensors has stopped before their full potential has been realised. EBCMOS cameras, on the other hand, are a recent development and not yet widely available, but show great potential, especially regarding the increased frame rate (500 Hz has been demonstrated [46]). A distinctive advantage of EB-sensors is the low dark count due to thermionic emission from the photocathode, in common with other photocathode and MCP-based devices, for which 0.02 events/s/cm^2^ have been quoted [63], This would be useful for situations where a good signal to noise ratio is required, e.g., for very weak bioluminescence, or decay measurements of probes with microsecond decay times, for example oxygen sensing, or time-resolved fluorescence anisotropy measurements of large molecular weight proteins for which nanosecond decay times are too short.

A voltage sweep could be used to time photon arrival in EB pixel image sensors. Gated intensifiers can operate with gates as short as 200 ps over ∼1.5 kV [64,65,66], and some gated optical intensifiers and high rate imagers, which have been used for time-gated FLIM for over a decade [67,68], can operate at 500 ps gate width at 100 MHz. If the EBCCD gain can be swept in 50 ns or so over 4 kV, this approach seems feasible. With a photoelectron time of flight of 25 ps at 8 kV and 35 ps at 4 kV according to Equation (3), the tens of nanosecond sweep times which would typically be needed for nanosecond fluorescence lifetime measurements are a thousand times longer than the time-of-flight of the photoelectron. The length of the time window, *i.e.*, sweep time, would also be easily adjustable, as in a TAC, by adjusting the duration of the voltage gain sweep: it could extend over microseconds to measure decays in that range [9].

Single pixel hybrid detectors, which comprise a photocathode in front of an avalanche photodiode (biased below the breakdown voltage), are excellent for photon arrival timing with picosecond resolution [21,22] and are often used in scanning fluorescence microscopy-based FLIM [69,70]. The single photoelectrons liberated by photons at the photocathode are accelerated across a high voltage (8 kV or so) into the avalanche photodiode. They can have a GaAsP photocathode with a high quantum efficiency of 50% around 500 nm, a large active area, are free of afterpulsing and cost less than a MCP.

SPAD arrays are extremely promising alternative devices, based on all-solid state sensors, to perform photon arrival timing in each pixel with picosecond resolution. At the time of writing, the fill factor (*i.e.*, the light sensitive area compared to the whole pixel area) is low, although this is being addressed by current developments in 3D stacking of integrated circuits [71]. Moreover, microlens arrays can be placed in front of the detector to focus more of the fluorescence signal onto the light-sensitive area [72]. The low fill factor problem can also be circumvented by multibeam scanning fluorescence microscopy, by projecting the fluorescence onto the light sensitive area only [73,74]. The noise levels of SPAD arrays are currently higher than for photocathode based detectors; the dark noise performance of SPAD arrays, typically 100s of counts per pixel (SPAD), depending on the operating voltage and temperature [75], can be improved to 10s of counts per pixel (25 Hz has been quoted [76]), and appropriate cooling could reduce this further. Nevertheless, the outstanding capability of enormous global count rates well into the gigahertz region [77] is a decisive advantage of these devices.

## 6. Conclusions

In electron-bombarded sensors a single photoelectron is accelerated directly into a CCD or CMOS sensor without multiplication. With a low gain, these devices can be used in analogue mode, and at high gain, they are sensitive enough to detect single photons: they enable wide-field imaging at extremely low light levels, allowing the detection of up to hundreds or thousands of photon events per frame. Photon event centroiding can be employed to recover resolution lost in the detection process, as described in more detail in [58]. Unlike photon counting cameras employing electron-multiplying MCPs, the photon events have a narrow, acceleration-voltage-dependent pulse height distribution. A gain voltage sweep during exposure in an EB-sensor could allow photon arrival time determination from the pulse height with sub-frame exposure time resolution. The low noise performance of EB-sensors may make them suitable for ultra-low intensity measurements, or time-resolved imaging of microsecond decay probes, e.g. for oxygen sensing, or for time-resolved fluorescence anisotropy measurements, or imaging of large molecular weight proteins.
